# Targeted Sequencing and RNA Assay Reveal a Noncanonical *JAG1* Splicing Variant Causing Alagille Syndrome

**DOI:** 10.3389/fgene.2019.01363

**Published:** 2020-01-24

**Authors:** Yiyao Chen, Xueli Liu, Songchang Chen, Junyu Zhang, Chenming Xu

**Affiliations:** ^1^International Peace Maternity and Child Health Hospital, School of Medicine, Shanghai Jiao Tong University, Shanghai, China; ^2^Shanghai Key Laboratory of Embryo Original Diseases, Shanghai, China; ^3^Shanghai Municipal Key Clinical Specialty, Shanghai, China

**Keywords:** *JAG1*, Alagille syndrome, targeted sequencing, RNA assay, prenatal diagnosis

## Abstract

Alagille syndrome (ALGS), as known as congenital arteriohepatic dysplasia, is a rare autosomal dominant multi-systemic disorder. Mutations in *JAG1* or more rarely *NOTCH2* have been reported as the cause of ALGS. In this study, a 5-year old girl with typical ALGS feature and her pregnant mother came to our reproductive genetics clinic for counseling. We aimed to clarify the genetic diagnosis and provide prenatal genetic diagnosis for the pregnant. Next generation sequencing (NGS)-based multigene panel was used to identify pathogenic variant of the proband. Then the candidate variant was verified by using Sanger sequencing. RNA assay was performed to clarify splicing effect of the candidate variant. Amniocentesis, karyotyping, and Sanger sequencing were performed for prenatal testing. We found a novel *de novo* noncanonical *JAG1* splicing variant (c.2917-8C > A) in the proband. Peripheral blood RNA assay suggested that the mutant transcript might escape nonsense-mediated messenger RNA (mRNA) decay (NMD) and encode a C-terminal truncated protein. Information of the variant has resulted in a successful prenatal diagnosis of the fetus. Our results clarified the genetic diagnosis of an ALGS patient and ensured utility of prenatal genetic testing.

## Introduction

Alagille syndrome (ALGS) is a multi-systemic congenital disorder characterized by paucity of intrahepatic bile ducts in combination with five primary clinical findings, inducing cholestasis, cardiac abnormalities, skeletal malformations, ocular abnormalities, and characteristic facial features (Li et al., 1997). The lack of bile duct directly causes cholestasis. In addition, about 39% of ALGS patients also have renal involvement, mainly renal hypogenesis (Kamath et al., 2012b). Current managements for ALGS patients are supportive in which most cases require lifetime surveillance and relevant operations. Besides, nearly 94% patients harbor congenital structural cardiac diseases and 21–31% patients require a liver transplantation (Mitchell et al., 2018). ALGS has a prevalence of approximately 1:70,000 based on the presence of neonatal liver disease (Vozzi et al., 2013).

Defects in components of the NOTCH signaling pathway are associated with ALGS (Turnpenny and Ellard, 2012). Currently, two genes have been revealed as the cause of ALGS. Approximately 94% of the ALGS patients are associated with loss-of-function variants of *JAG1* gene, subclassified as ALGS1 (OMIM 118450); while 1–2% of patients are due to *NOTCH2* gene, subclassified as ALGS2 (OMIM 610205) (Mcdaniell et al., 2006; Kamath et al., 2012a). The phenotypic expression and penetrance of *JAG1/NOTCH2* variants are variable (Grochowski et al., 2016). There is no strong correlation between the genotype and phenotype, suggesting that other genetic modifiers beyond the known variants may be the cause of the variable expressivity of ALGS. ALGS is inherited in an autosomal dominant pattern with about 50–70% of cases have *de novo* pathogenic variants. The risk of sibs of a proband who with an apparent *de novo* pathogenic variant is very low, but still greater than the general population because of the possibility of germline mosaicism (Campbell et al., 2014).

*JAG1* gene, located on chromosome 20p12.2, consists of 26 exons and encodes a ligand for the NOTCH receptor. The *JAG1* domains physically interact with NOTCH2 and other NOTCH factors to trigger cascading proteolytic cleavages, leading to transport of the intracellular domain of NOTCH receptor into the nucleus, and activation of transcription factors related to cell differentiation and morphogenesis (Grochowski et al., 2016). To date, over 692 *JAG1* mutations have been reported in HGMD (the Human Gene Mutation Database) (Stenson et al., 2003).

The rapid development and application of next-generation sequencing (NGS) enables the screening of the entire exome/genome and reveals an increasing amount of new pathogenic causes of genetic diseases. In this study, we identified a novel *de novo* noncanonical splicing *JAG1* pathogenic variant as molecular cause in a Chinese ALGS family. The effect of the variant was analyzed using RNA assay. Additionally, the potential risk of transmitting the variant was prenatally diagnosed using fetal DNA derived from amniocytes.

## Materials and Methods

### Subjects and Ethics Statement

Peripheral blood samples (including the family members and 592 ethnically matched unrelated controls) for the study were recruited from the International Peace Maternity & Child Health Hospital (IPMCH), Shanghai Jiao Tong University School of Medicine. Genomic DNAs were isolated from peripheral blood according to standard procedures. This study was conducted in accordance with the Declaration of Helsinki and approved by the Ethics Review Committee of IPMCH. Informed consents were obtained from all participants or their legal guardians.

### Targeted Sequencing and Data Analysis

DNA sample obtained from the proband (II-1) was sequenced using targeted next generation sequencing. A 2,181-gene medical exome panel was used for exomes and flanking sequence capture. The procedure for preparation of library was published previously (Liu et al., 2015; Zhang et al., 2017). Sequencing of the captured target regions was performed paired-end sequencing (2×90bp) on Illumina HiSeq2500 Analyzer (Illumina, San Diego, CA, USA). Clean sequencing reads were aligned to the human reference genome GRCh37/hg19 by Burrows-Wheeler Aligner (BWA; v.0.7.12) (Li and Durbin, 2009). Variant calling and annotation were performed using the Genome Analysis Toolkit (GATK; version 3.5) and ANNOtate VARiation (ANNOVAR) (Yang and Wang, 2015), respectively. The resulting variants were prioritized with our in-house developed pipeline (MultiOmics One) (Zhang et al., 2017). The interpretation of variants was based on the American College of Medical Genetics and Genomics and the Association for Molecular Pathology (ACMG/AMP) guideline (Richards et al., 2015).

### Variant Confirmation and RNA Assay

Prioritized variants were confirmed by Sanger sequencing. The primers for verifying *JAG1* variant (*JAG1*-F: 5'-ggatgtctgcttgcttgctt-3' and *JAG1*-R: 5'-gaactgccttgccatcgaat-3') were designed by Primer 3 online (Untergasser et al., 2012). Variant nomenclature was based on the Human Genome Variation Society (HGVS) naming conventions (Den Dunnen et al., 2016).

For RNA assay, total RNAs were extracted and reversely transcribed from peripheral blood mononuclear cells (PBMCs) of the proband (II-1) and a healthy control. The Reverse transcription (RT)-PCR were performed with the primers *JAG1*-E22F: 5'-CAGATGGGGCCAAATGGGAT-3' (exon 22) and *JAG1*-E26R:5'-GCTCAGCAAGGGAACAAGGA-3' (exon 26). The amplified DNA fragments were analyzed using Qsep100 DNA Analyzer (BiOptic Inc., Taiwan, China) and subcloned into the pGM-T vector (TIANGEN Biotech, Beijing, China) for sequencing.

### Prenatal Testing and Short Tandem Repeat Identity Testing

Amniocentesis was performed in 17-week of gestation under continuous ultrasonographic guidance (Practice Bulletin No. 162: Prenatal Diagnostic Testing for Genetic Disorders, 2016). Thirty milliliters of amniotic fluid sample was obtained. Chromosomal karyotyping and Sanger sequencing were conducted in parallel in both cultured and uncultured samples. Identity testing was performed using the human personal identification detection kit (R1004T; GENESKY, Shanghai, China) for *de novo* variant confirmation and potential maternity contamination analysis. The PCR amplicons were analyzed using Applied Biosystems 3500Dx sequencer.

## Case Presentation

**Figure 1:**
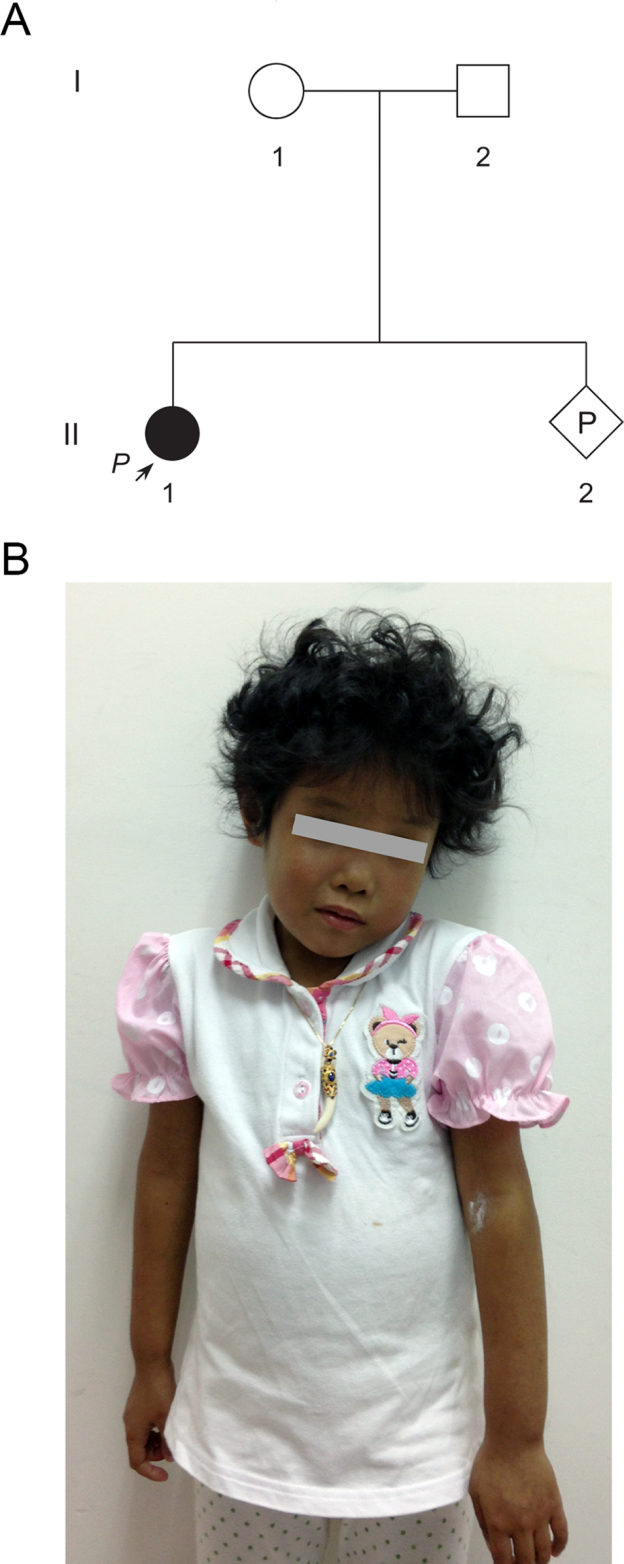
Pedigree of a Chinese family at-risk for recurrence of Alagille syndrome (ALGS). **(A)** Arrow indicates the proband. All family members were subjected to genetic analysis. **(B)** The proband was presented with jaundice skin and curly hair.

A 33-year-old pregnant woman of 9-week gestation was referred to our reproductive genetics clinic for counseling (**Figure 1A**). The 5-year-old proband (II-1) was born at full term with low birth weight (2,162 g). She was presented with jaundice in skin and sclera shortly after birth to the present (**Figure 1B**). Echocardiography showed an atrial septal defect. Other features include hepatosplenomegaly and curly hair (**Figure 1B**). Laboratory testing done at that time revealed raised cholestasis makers, including total bilirubin (TBIL) 231.1 μmol/L (normal range 5.1–17.1 μmol/L), direct bilirubin (DBIL) 187.6 μmol/L (normal range 0–6 μmol/L), total bile acid (TBA) 99.7 μmol/L (normal range 0–10 μmol/L), γ-glutamyl transpeptidase (GGT) 174 IU/L (normal range 7-50 IU/L), aspartate aminotransferase (AST) 81 IU/L (normal range 0–40 IU/L), and alkaline phosphatase (ALP) 876 IU/L (normal range 42–383 IU/L). Further tests showed normal values of vitamin D, vitamin E, and prothrombin time (PT). The clinical features of the patient suggested a diagnosis of Alagille syndrome.

## Results

### Identification of a Novel *JAG1* Splicing Variant by Target Sequencing

While the clinical characteristics supported a diagnosis of ALGS, genetic analysis of the proband revealed no pathogenic variants in *JAG1* or *NOTCH2* in another clinical laboratory previously. To search for genetic variant that might explain the patient's clinical findings, NGS-based targeted sequencing of 2,181 genes related to Mendelian disorder of digestive system was performed using genomic DNA of the proband (II-1). The average sequencing depth of the target region is 374.47× with 97.92% of bases reached at least 30× coverage. Date priority filtering revealed that the proband was heterozygous for a novel noncanonical splicing variant of NG_007496.1(JAG1):c.2917-8C > A in splicing consensus region of 3' splice site (Prioritized variants are listed in **Table S3**).

**Figure 2:**
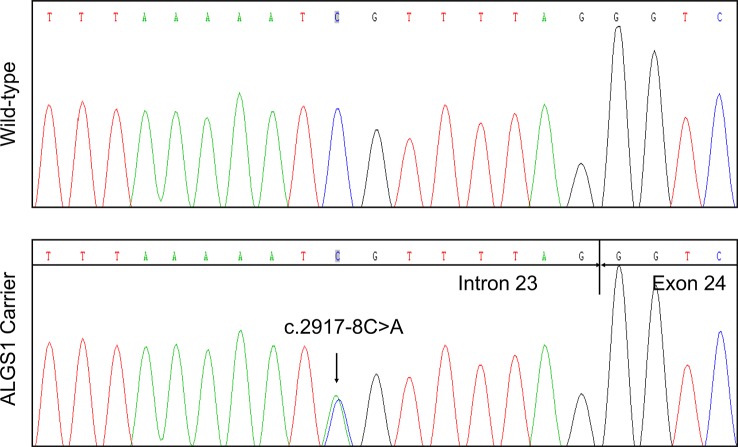
Identification of a novel *JAG1* pathogenic variant in the proband. Sanger sequencing verified the next generation sequencing (NGS) result. The arrows indicate the alteration from C to A.

In agreement with the NGS result, Sanger sequencing confirmed the proband was heterozygous for the variant (**Figure 2**). The variant was not detected in other family members. Subsequent identity testing using short tandem repeat (STR) markers revealed the *de novo* feature of the variant (**Table S1**). The variant was neither found in 592 unrelated control subjects, nor included in public population databases, including genome Aggregation Database (gnomAD; http://gnomad.broadinstitute.org) (Lek et al., 2016), Exome Aggregation Consortium (ExAC; http://exac.broadinstitute.org) (Lek et al., 2016), 1000 Genomes Project (http://browser.1000genomes.org) (Genomes Project et al., 2015).

### The *JAG1* c.2917-8C > A Variant Generated an Aberrant Transcript Retained Partial Intron Sequence

**Figure 3:**
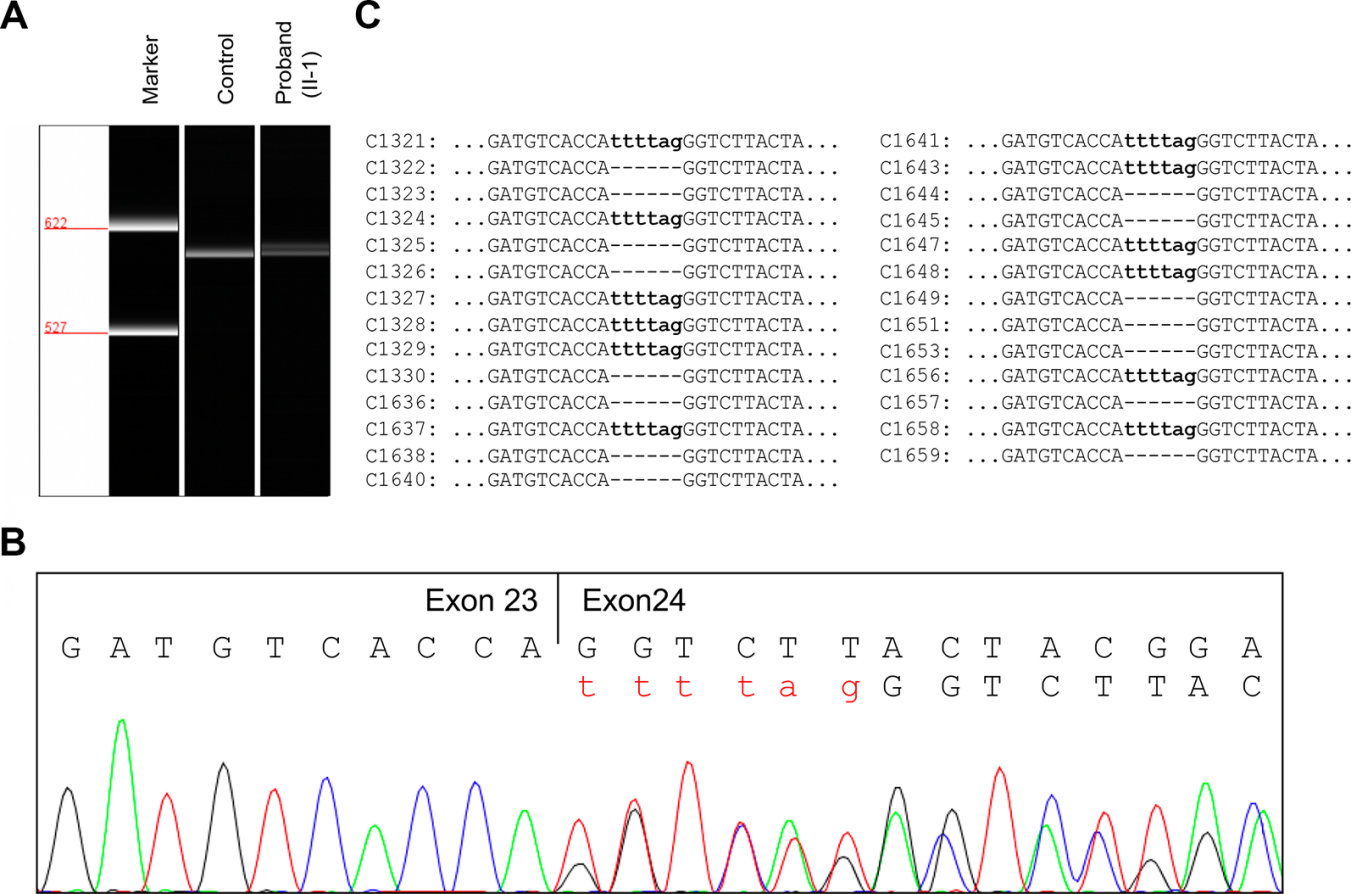
RNA assay of the *JAG1* messenger RNA (mRNA). **(A)** Reverse transcription (RT)-PCR analysis of *JAG1* mRNA from peripheral blood mononuclear cells (PBMCs) of the proband (II-1) and a healthy female control. The complementary DNA (cDNA) fragments of 611-bp were identified in both the proband and the wild-type control. In addition, a little larger fragment was detected in the proband, but not in the wild-type. **(B)** cDNA sequencing revealed that *JAG1* transcripts of the proband were heterozygous for c.2917-8C > A. **(C)** Sequencing analysis results of the pGM-T clones with the cDNA amplicons.

To evaluate the effect of the splicing variant, *in silico* splicing assays were performed. While Human Splicing Finder (HSF 3.0) (Desmet et al., 2009) predicted splice acceptor site in wild-type *JAG1*, the activation of an intronic cryptic acceptor site was suggested in the presence of c.2917-8C > A. RNA assay was then performed to confirm the predicted result. RT-PCR analysis of RNA extracted from peripheral blood mononuclear cells (PBMCs) of the proband and a healthy control was carried out using primers located at exon 22 and exon 26. The proband and healthy control showed a different splicing pattern (**Figure 3A**).

Further complementary DNA (cDNA) sequencing indicated that the *JAG1* transcripts of the proband were heterozygous for r.2916_2917ins2917-6_2917-1 (**Figure 3B**). This result suggested the variant could destroy the intron23/exon 24 splice acceptor site and active a new stronger acceptor site in intron 23 (position c.2917-7/c.2917-6). The intron 23 sequence from positions c.2917-6 to c.2917-1 (TTTTAG) was inserted in the mutant transcript, consequently. Thus, the splicing variant could be predicted to introduce a premature termination codon (PTC) in position 974, p.(Gly973Phefs*2). The putative PTC is located 283-bp upstream of the last exon 25-exon 26 junction; thus the corresponding mutant transcript should not escape the nonsense-mediated mRNA decay (NMD) (Maquat, 2004; Khajavi et al., 2006).

To further investigate the influence of the NMD on *JAG1* mutant transcript, RT-PCR products were then subcloned into pGM-T vector. Totally, 27 clones were sequenced using T7 universal primer. Sequence analysis showed a 15:12 proportion of wild-type and r.2916_2917ins2917-6_2917-1 clones (**Figure 3C**). Together, these results suggested the *JAG1* c.2917-8C > A variant could generate an aberrant transcript, which might escape the NMD and encode a C-terminal truncated protein.

### The Variant Was Classified to Pathogenic Variant and Prenatal Tested in the Pregnant

The *de novo* variant was classified as “pathogenic” according the PS2, PS3, PM2, PP3, and PP4 criteria of the ACMG/AMP guidelines (Richards et al., 2015). The family was counseled with low recurrence risk, but that gonadal mosaicism could not be excluded. The amniocentesis was carried out in 17-week of gestation of the proband's mother. Variant analysis and chromosomal karyotyping (**Figure S1**) results showed the fetus (II-2) was unaffected with normal female karyotype. STR markers analysis confirmed that the sample on testing was not contaminated by maternal blood (**Table S1**). The pregnancy was continued and delivered at 39 weeks' gestation with the Apgar score of 10-point.

## Discussion

ALGS is an autosomal dominant multi-system disorder which is caused by pathogenic variants in *JAG1* or *NOTCH2*. Only a few cases of ALGS were reported in Chinese population (Li et al., 2015). Published articles reveal a wide spectrum of phenotypes ranging from cardiac or hepatic manifestations and intracranial bleeding associated with morbidity and mortality to subclinical symptoms, thus a genetic diagnosis can be useful for atypical patients. The clinical diagnosis and management of ALGS patients require a multidisciplinary panel of experts. Despite therapeutic regimens like choleretic agents and biliary diversion prove to be helpful to relieve the symptoms; ongoing surveillance of growth, diet, nutritional conditions, and multi-organ functions is demanded in the lifetime. Genetic counseling is recommended to families with an ALGS patient and in want of a healthy child.

In our study, it's interesting to highlight that we identified a noncanonical splicing *JAG1* variant located at −8 bp from intron 23/exon 24 boundary using our pipeline. However, the PVS1 evidence could only be used for the canonical ±1 or 2 splice sites according to the ACMG/AMP guidelines (Richards et al., 2015). The default setting of ANNOVAR also merely marked ±1 or 2 splice sites as splicing variant (Grochowski et al., 2016). These may contribute to the underdiagnosis of the proband by the other clinical laboratory. Functional analysis for splicing variant is essential. Besides the variant identified in our study, the other variant of c.2917-8C > T, at the same position of our variant, has been identified in two individuals with an allele frequency of 0.000007974 in gnomAD. However, the Human Splicing Finder prediction results only supported a deleterious effect on our variant. Most importantly, further RNA assay and clone sequencing data proved the splicing alteration of our variant. Three noncanonical splicing variants near the c.2917-8C > A variant, including c.2917-13 _2917-8del (Warthen et al., 2006), c.2917-5_2919dup (Pilia et al., 1999), c.2917-10A > G (Stalke et al., 2018), have been reported in patients with ALGS (**Figure S2**, **Table S2**). The c.2917-10A > G variant is most similar to the one reported in our study. The variant was reported as “variant of uncertain significance,” although it's identified in a typical ALGS patient, and the *in-silico* predictions suggested deleterious affecting on splicing. Further RNA assay on this variant might upgrade the classification to “likely pathogenic” or “pathogenic.” Together, our experience shows that detailed analysis of variants that may affect splicing at noncanonical position can provide a definite molecular diagnosis when the clinical manifestations clearly suggest the involvement of a set of candidate genes, but the standard (sequencing) techniques do not provide a clear answer.

To date, the guideline for pregnancy management in families with ALGS patients has not been established in terms of its low prevalence. Prenatal genetic testing for couples at-risk is possible if the pathogenic variant in the proband is known. In this study, amniocentesis, karyotyping, and Sanger sequencing were performed for the pregnant mother, and a healthy baby girl was delivered at 39 weeks' gestation. Approximately 50–70% of ALGS cases are caused by *de novo* pathogenic variant. The mosaicism frequency for ALGS cases has been reported more than 8.2%, which should be taken seriously in genetic counseling (Giannakudis et al., 2001). For parents of a child with an apparent *de novo* pathogenic variant, recurrence risk to subsequent offspring with ALGS is low but still greater than in the general population because of the possibility of germline mosaicism.

In conclusion, we identified a novel noncanonical splicing *JAG1* pathogenic variant (c.2917-8 C > A) in an ALGS patient by using multigene panel testing. Furthermore, peripheral blood RNA assay was performed and indicated that the mutant transcript could escape NMD and might encode a C-terminal truncated protein. Moreover, we also led to a successful prenatal diagnosis of the pregnant mother. The novel pathogenic variant could enrich the mutation database of *JAG1*. Our approach of functional analysis splicing variant would provide valuable insight into clarifying those of noncanonical splicing variants.

## Data Availability Statement

The raw data supporting the conclusions of this article will be made available by the authors, without undue reservation, to any qualified researcher.

## Ethics Statement

The studies involving human participants were reviewed and approved by Ethics Review Committee of the International Peace Maternity & Child Health Hospital. The patients/participants provided their written informed consent to participate in this study. Written informed consent was obtained from the minor(s)' legal guardian/next of kin for the publication of any potentially identifiable images or data included in this article.

## Author Contributions

JZ and CX conceived the study. JZ and SC collected the data. YC and JZ conducted the experiment. YC and JZ participated in the data analysis. YC, XL, and JZ wrote the draft manuscript. JZ and CX revised the manuscript.

## Funding

This work is supported by the National Key R&D Program of China [NO. 2016YFC0905103], National Natural Science Foundation of China [NO. 81771638, 81471506, 81401219], the Science and Technology Commission of Shanghai Municipality [NO.19ZR1462300, 17411972900, 15411966700, 15411964000], the Municipal Human Resources Development Program for Outstanding Young Talents in Medical and Health Sciences in Shanghai [NO. 2018YQ39], the Shanghai Jiao Tong University Program [NO. YG2017MS39].

## Conflict of Interest

The authors declare that the research was conducted in the absence of any commercial or financial relationships that could be construed as a potential conflict of interest.
