# Supplementary material for: Targeted Sequencing and RNA Assay Reveal a Noncanonical JAG1 Splicing Variant Causing Alagille Syndrome
Source: Front Genet. 2020 Jan 24;10:1363. doi: 10.3389/fgene.2019.01363 (PMC6993058; doi:10.3389/fgene.2019.01363)

Figure S1. Prenatal diagnose of the fetus. (A) Sanger sequencing indicated that the fetus was unaffected. (B) Karyotyping result of the fetus was a normal 46,XX chromosomal constitution.

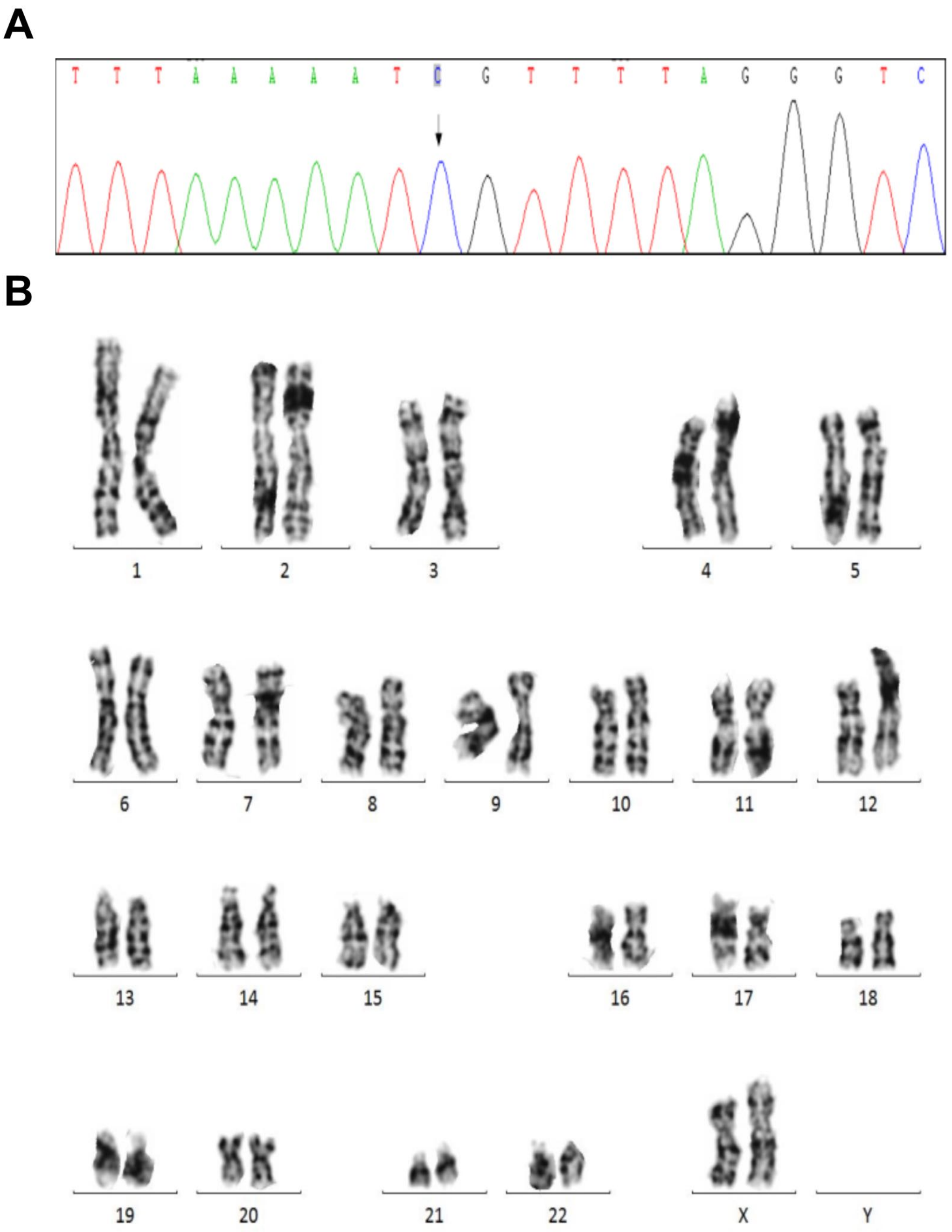

Supplement: Supplementary file 1 [file Image_1.pdf]
