# Supplementary material for: Targeted Sequencing and RNA Assay Reveal a Noncanonical JAG1 Splicing Variant Causing Alagille Syndrome
Source: Front Genet. 2020 Jan 24;10:1363. doi: 10.3389/fgene.2019.01363 (PMC6993058; doi:10.3389/fgene.2019.01363)

Figure S2. Schematic representation of noncanonical splicing variants reported in *JAG1*. The NM_000214.2 transcript was used. Exons (in black), introns (lines), and UTR (in grey) are represented.

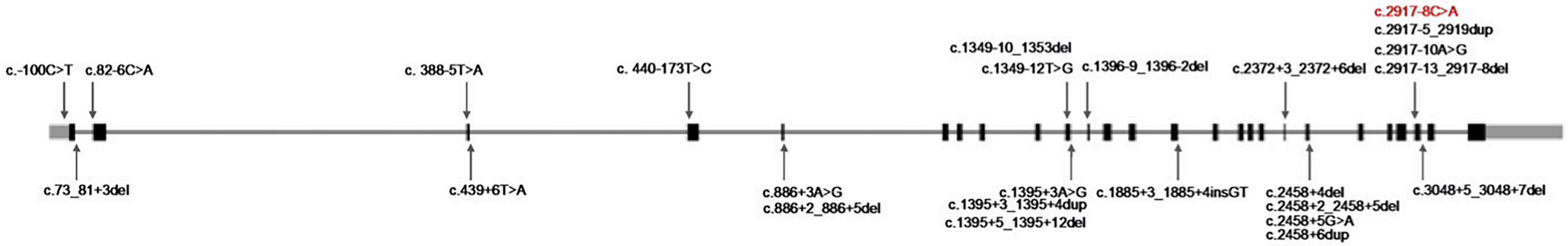

Supplement: Supplementary file 2 [file Image_2.pdf]
