# Supplementary material for: Targeted Sequencing and RNA Assay Reveal a Noncanonical JAG1 Splicing Variant Causing Alagille Syndrome
Source: Front Genet. 2020 Jan 24;10:1363. doi: 10.3389/fgene.2019.01363 (PMC6993058; doi:10.3389/fgene.2019.01363)
Supplement: Supplementary file 3 [file Table_1.pdf]

Table S1. Short tandem repeat typing results for the members of the pedigree.

Marker	Ⅰ-1		Ⅰ-2		Ⅱ-1		Ⅱ-2	
	Allele 1	Allele 2	Allele 1	Allele 2	Allele 1	Allele 2	Allele 1	Allele 2
Amelo	X	Y	X	X	X	X	X	X
D13S317	8	8	11	11	8	11	8	11
D7S820	11	12	11	12	11	12	12	12
G4S0001	14	16	14	15	14	14	14	15
G2S0002	16	19	16	20	19	20	16	19
D18S51	14	20	14	14	14	20	14	14
D8S1179	10	14	13	15	10	15	10	14
D2S1338	17	23	19	23	17	23	17	19
G15S0001	14	14	12	13	12	14	12	14
D16S539	13	13	12	12	12	13	12	13
VWA	14	14	14	18	14	18	14	14
G7S0005	10	11	10	11	10	11	10	10
G10S0001	18	18	18	20	18	20	18	20
THO1	9	9	7	9	9	7	9	9
D8S588	11	12	10	12	10	12	10	12
G5S0001	7	10	7	11	10	11	7	10
D5S818	10	13	9	12	9	10	10	12

Note: The analysis was performed by a human identification kit of the 16 short tandem repeat loci and amelogenin. Values shown in the chart indicated the numbers of short tandem repeats at each allele of each locus.
