# Supplementary material for: Targeted Sequencing and RNA Assay Reveal a Noncanonical JAG1 Splicing Variant Causing Alagille Syndrome
Source: Front Genet. 2020 Jan 24;10:1363. doi: 10.3389/fgene.2019.01363 (PMC6993058; doi:10.3389/fgene.2019.01363)
Supplement: Supplementary file 4 [file Table_2.pdf]

Table S2. Noncanonical splicing variants reported in *JAG1*

Variant	Reference
c.-100C>T	Stalke (2018) Clin Genet 93,665
c.73_81+3del	Heritage (2000) Hum Mutat 16,408
c.82-6C>A	Vazquez-Martinez (2014) Meta Gene 2,32
c.388-5T>A	Crosnier (1999) Gastroenterology 116,1141
c.439+6T>A	Warthen (2006) Hum Mutat 27,436
c.440-173T>C	Kung (2010) Am J Hum Genet 86,229
c.886+3A>G	Heritage (2002) Hum Mutat 20,481
c.886+2_886+5del	Jurkiewicz (2014) J Appl Genet 55,329
c.1349-12T>G	Krantz (1998) Am J Hum Genet 62,1361
c.1349-10_1353del	Li (2015) PLoS One 10,130355
c.1395+3A>G	Pilia (1999) Hum Mutat 14,394
c.1395+3_1395+4dup	Bhatia (2014)Indian Pediatr 51,314
c.1395+5_1395+12del	Heritage (2000) Hum Mutat 16,408
c.1396-9_1396-2del	Crosnier (1999) Gastroenterology 116,1141
c.1885+3_1885+4insGT	Li (2015) PLoS One 10,130355
c.2372+3_2372+6del	Crosnier (1999) Gastroenterology 116,1141
c.2458+4delA	Guegan (2012) Clin Genet 82,33
c.2458+2_2458+5del	Crosnier (1999) Gastroenterology 116,1141
c.2458+5G>A	Warthen (2006) Hum Mutat 27,436
c.2458+6dup	Yuan (2001) Clin Genet 59,330
c.2917-10A>G	Stalke (2018) Clin Genet 93,665
c.2917-13_2917-8del	Warthen (2006) Hum Mutat 27,436
c.2917-5_2919dup	Pilia (1999) Hum Mutat 14,394
c.3048+5_3048+7del	Ropke (2003) Hum Mutat 21,100
